# Characterization of the complete chloroplast genome of *Chasmanthium latifolium* (Michx.) H.O.Yates, 1966 (Poaceae)

**DOI:** 10.1080/23802359.2025.2460781

**Published:** 2025-02-02

**Authors:** Ge Fang, Hao Jiang, Jinting Zhang, Ren Wang, Xiangdong Chen, Tiezhu Hu, Xiaojun Wu, Zhengang Ru

**Affiliations:** a Center of Wheat Research, Henan Institute of Science and Technology, Xinxiang, China; b School of Agriculture, Henan Key Laboratory of Hybrid Wheat, Xinxiang, China; c Henan Collaborative Innovation Center of Modern Biological Breeding, Xinxiang, China

**Keywords:** *Chasmanthium latifolium*, gramineae, chloroplast genomics, phylogenetic analysis

## Abstract

*Chasmanthium latifolium* (Michx.) H.O.Yates is a popular ornamental plant native to southeastern North America. Genomic data and genetic studies related to *Chasmanthium latifolium* are limited. Therefore, the complete chloroplast genome of *Chasmanthium latifolium* was sequenced, assembled, and characterized in this study. The complete chloroplast genome was 138,934 bp in length and contained 105 unique genes (77 protein-coding genes, 24 tRNA genes, and 4 rRNA genes). Phylogenetic analyses showed that *Chasmanthium latifolium* and *Chasmanthium laxum* clustered into a separate clade with the closest affinity to the clade comprising *Zeugites pittieri* Hack and *Lophatherum gracile* Brongn. In conclusion, our study describes the complete chloroplast genome of *Chasmanthium latifolium* for the first time, contributing to a better understanding of its taxonomy and evolution.

## Introduction

*Chasmanthium latifolium* (Michx.) H.O.Yates (2n = 48), commonly known as Spangle Grass, River Oats, or Sea Oats, is a perennial grassy herbaceous ornamental plant native to wet woodlands and forests in southeastern North America (Tucker 1990; Harvey and Brand 2002; Liliana 2013). *C. latifolium* prefers moist, fertile soils that are soil-adapted, salt-tolerant, and tolerant of both drought and moisture; it grows readily in moist well-drained soils and is fairly tolerant of shade (Darke 1994; Cole and Cole 2000) and can spread by rhizomes and seeds (Yates1966; Keck et al. 2014). Studies have shown that *C. latifolium* is a shade-adapted C3 species, generally growing between 0.5 and 1.2 m in height, with distinctive flat nodding seed heads similar to that of oats and is a food source for small mammals and birds (Nebhut et al. 2022).

The chloroplast genome is matrilineally inherited, with a clear pattern of inheritance; it is relatively conservative, with stable gene order and structure, which makes it easy to compare between different species; and it is rich in genes with a moderate degree of sequence variation. Therefore, it is advantageous to be used in taxonomic classification and phylogenetic study of plant species (Clegg et al. 1994; Zhang et al. 2016). To date, chloroplast genome data for the genus *Chasmanthium* have been reported only for *Chasmanthium laxum* (L.) H.O.Yates, which limits phylogenetic and evolutionary analyses of the genus and its related taxa (Burke et al. 2016; Teisher et al. 2017). Therefore, we publish the complete chloroplast genome data of *C. latifolium* for the first time with corresponding annotations and evolutionary analyses in this paper, and this study provides an important reference for insight into the taxonomic and evolutionary status of the genus and its related taxa, as well as the subsequent in-depth studies.

## Materials and methods

### Plant materials

**Figure 1.**
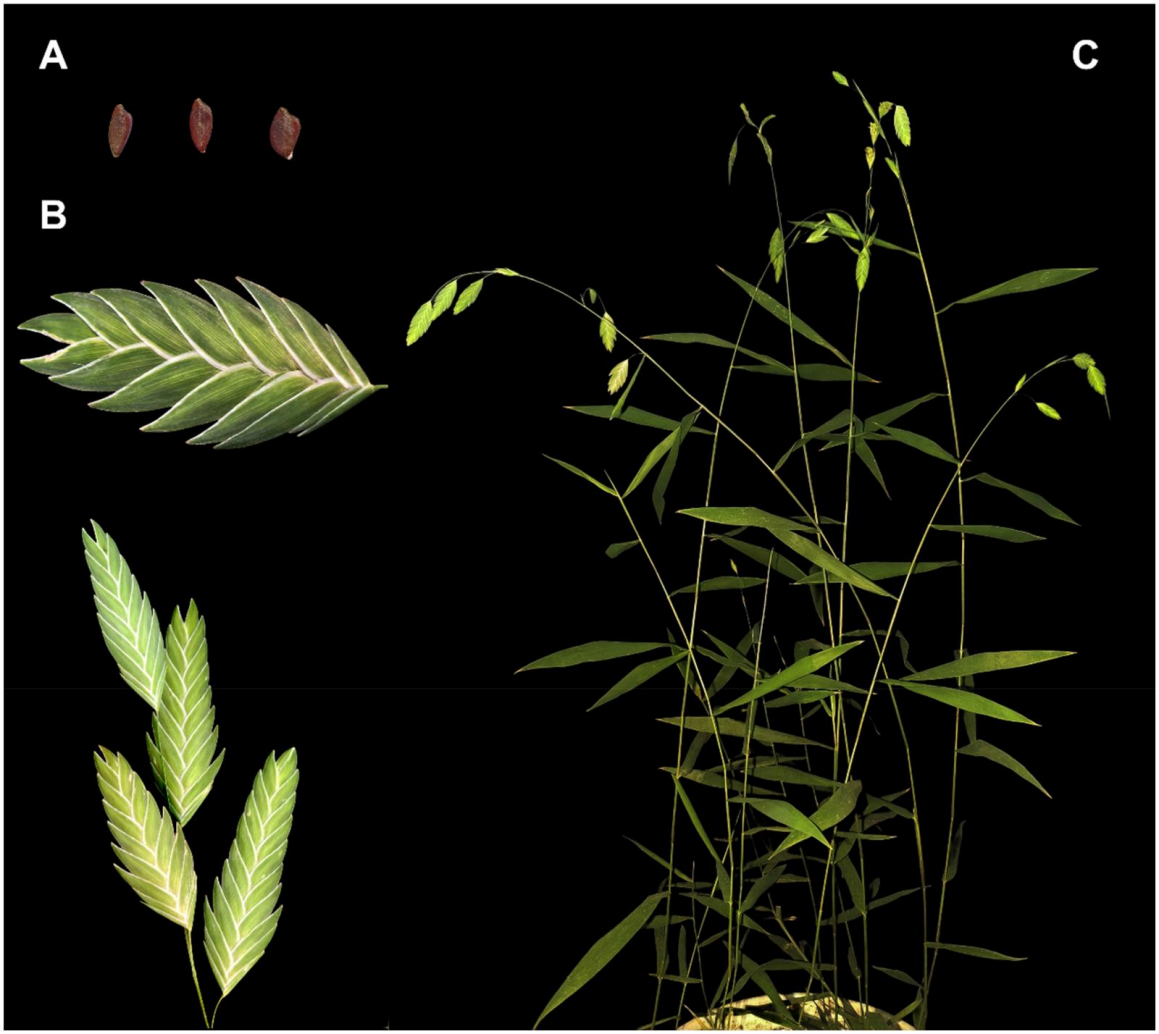
Morphology features of *Chasmanthium latifolium*. (A) mature seeds, reddish-brown, flattened, subelliptic; (B) inflorescence, panicle, flowering stems arcuate, with numerous spikes, spikelets broadly ovate, flattened and pendulous; (C) individual of *Chasmanthium latifolium*, stems erect, leaves striped, flattened. The photos of *Chasmanthium latifolium* were taken by Xiaojun Wu at the Botanical Garden of the Center of Wheat Research of Henan Institute of Science and Technology, Xinxiang, Henan, China.

Fresh leaves of *C. latifolium* were collected from the Botanical Garden of Wheat Research Center (113°88′E 35°30′N), Xinxiang City, Henan Province, China. The voucher specimen (contact: Zhengang Ru; rzgh5819@163.com) was deposited at the Herbarium of Henan Institute of Science and Technology, Xinxiang, China under the voucher number XM-W020 (Figure 1).

### DNA sequencing, genome assembly, and annotation

Total genomic DNA was extracted from clean leaves from samples frozen at −80 °C using the E.Z.N.A^®^ Plant DNA kit (OMEGA, Beijing). Genomic DNA was quantified by using a TBS-380 fluorometer (Turner BioSystems Inc., Sunnyvale, CA). High qualified DNA sample (OD260/280 = 1.8 ∼ 2.0, > 6ug) was utilized to construct the fragment library.

For NGS paired-end sequencing of samples, sequencing library construction was performed using the TruSeq^™^ Nano DNA Sample Prep Kit (Illumina, USA). The sequencing of the DNA library was performed on the Illumina NovaSeq 6000 platform (150 bp × 2, Shanghai BIOZERON Biotech. Co., Ltd), with coverage assessment carried out through the samtools depth utility. The raw paired-end reads were trimmed and quality controlled by Trimmomatic with parameters (SLIDINGWINDOW:4:15 MINLEN:75) (version 0.36, http://www.usadellab.org/cms/index.php?page=trimmomatic) (Bolger et al. 2014). Clean data obtained by the above quality control processes were used to do further analysis. The high-quality sequence data (7.36 Gb) using the assembler GetOrganelle v1.7.5 (https://github.com/Kinggerm/GetOrganelle) to assemble the complete chloroplast genome from scratch (Bankevich et al. 2012; J et al. 2020). Based on the above assembly steps, we obtained a master circle of the *C. latifolium* chloroplast genome.

The chloroplast genes were annotated using the online GeSeq tool (https://chlorobox.mpimp-golm.mpg.de/geseq.html), using the default parameters to predict protein-coding genes (PCGs), transfer RNA (tRNA), and ribosome RNA (rRNA) genes (Tillich et al. 2017). The position of each coding gene was determined using BLAST searches against reference chloroplast genes. Manual correction of start/end codon and intron/exon boundaries of genes was performed in the SnapGene viewer (https://www.snapgene.com/snapgene-viewer) by referring to the chloroplast genome *Chasmanthium laxum* (Burke et al. 2016; Teisher et al. 2017). The circular chloroplast genome map of *C. latifolium* was drawn using the OrganellarGenomeDRAW (http://ogdraw.mpimp-golm.mpg.de/cgi-bin/ogdraw.pl) (Lohse et al. 2007). Simple sequence repeats (SSRs) of the chloroplast genome of *C. latifolium* were determined by MISA (https://webblast.ipk-gatersleben.de/misa/).

### Phylogenetic tree construction

To determine the molecular position of *C. latifolium* with other closely related species, phylogenetic analyses were performed using a total of 53 complete chloroplast genome sequences. *Triticum aestivum*(*Triticum aestivum* cultivar Chinese Spring TA3008 and *Triticum aestivum* isolate lunxuan987)、*Oryza sativa* (*Oryza sativa* Japonica Group and *Oryza sativa* Indica Group) were used as outgroups. The chloroplast genome sequences were aligned using the online version of MAFFT v7 (https://mafft.cbrc.jp/alignment/server/) with default parameters (Katoh et al. 2019) and then performed the maximum likelihood (ML) phylogenetic analysis using PhyML 3.0 with the following setting: substitution model = GTR, bootstrap = 1000 (http://www.atgc-montpellier.fr/phyml/) (Guindon et al. 2010).

## Results

**Figure 2.**
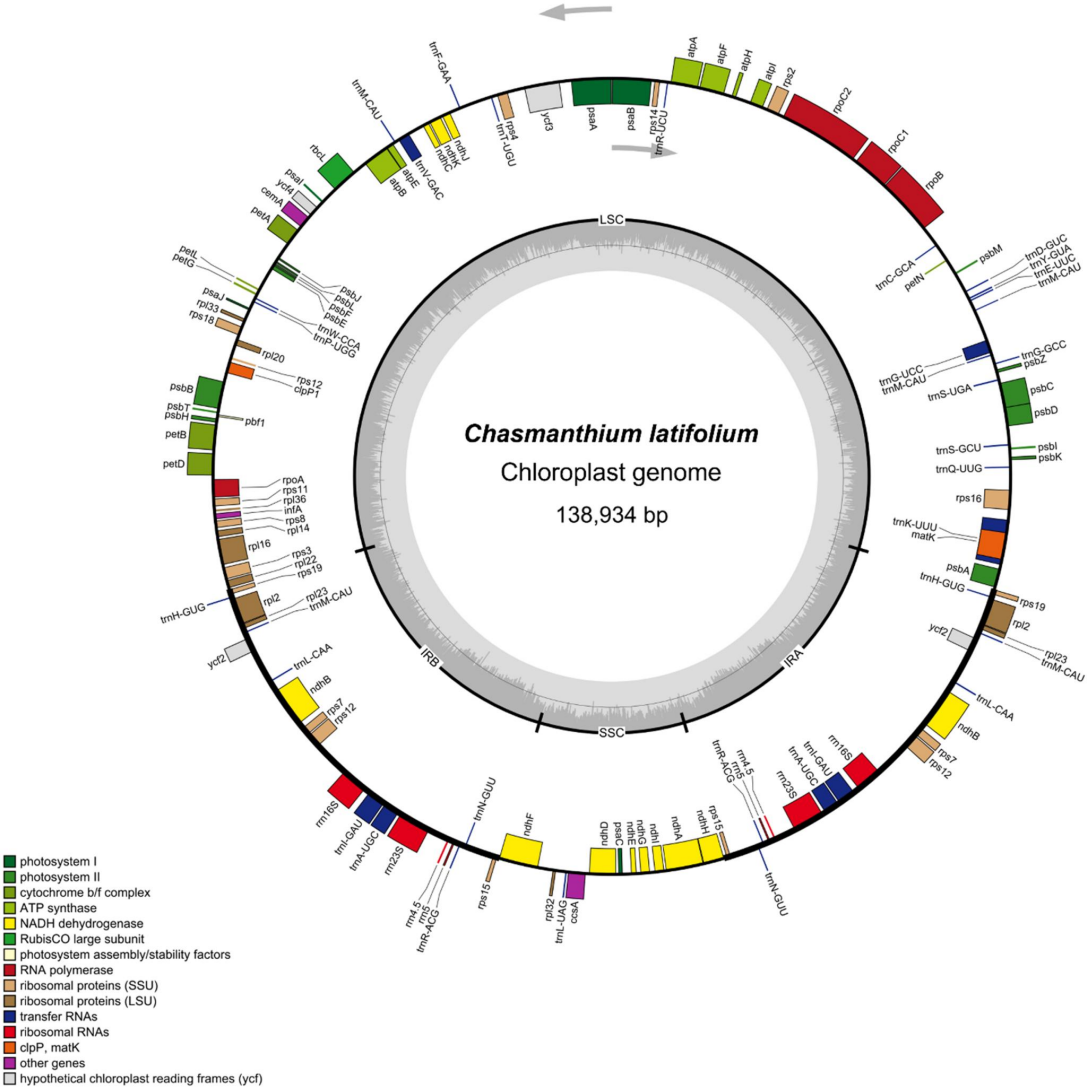
Chloroplast genome map of *Chasmanthium latifolium*. The assembled chloroplast genome (138,934 bp) of *Chasmanthium latifolium* (GenBank ID: PQ153239.1) with major features: there are 77 protein-coding genes, 24 tRNA genes, and four rRNA genes. The gray arrow inside the circle represents the transcriptional direction (clockwise) of the gene within the circle. The gray arrow outside the circle represents the transcriptional direction (counterclockwise) of the gene outside the circle. The light gray line inside the circle represents AT content, and the dark gray area represents GC content. At the bottom, the genes of different functional groups are indicated by various colors. This map was drawn using the OGDraw tool.

The assembled and annotated *C. latifolium* chloroplast genome has been uploaded to GenBank and obtained the accession number PQ153239.1. The *C. latifolium* chloroplast genome is 138,934 bp in length with a 2476 × depth of coverage (Figure S1) and exhibits a typical quadripartite structure consisting of a pair of inverted repeats (IRs) regions (22,070 bp) divided by a small single-copy (SSC) region (12,624 bp) and a large single-copy (LSC) region (82,170 bp) (Figure 2). Of the PCGs, 10 were cis-spliced genes, nine of which contained one intron, and one contained two introns (Figure S2a). In addition, the location of the exotic region of the trans-spliced gene rps12 was identified (Figure S2b). The GC content of the chloroplast genome was 38.78%, and the GC contents of the components IR, LSC, and SSC were 44.10%, 33.25%, and 36.77%, respectively.

A total of 105 genes were annotated in the *C. latifolium* chloroplast genome, including 77 PCGs, 24 tRNA genes, and 4 rRNA genes (Table 1). 31 genes were duplicated, including 17 PCGs, 10 tRNA genes, and 4 rRNA genes. It is worth mentioning that the *ycf1* gene was missing in the chloroplast genome of *C. latifolium*. In addition, 42 simple sequence repeats (SSRs) were found in the chloroplast genome of *C. latifolium*, including 24 mononucleotide repeats at most, 5 dinucleotide repeats, 4 trinucleotide repeats, and 9 tetranucleotide repeats (Figure S3).

**Table 1.** List of genes was annotated in the chloroplast genome of *Chasmanthium latifolium.*

Functional category	Groups of genes	Name of genes	No. of genes
Genes for photosynthesis	Subunits of photosystem I	*psa*A *psa*B *psa*C *psa*I *psa*J	4
	Subunits of photosystem II	*pbf1 psb*A *psb*B *psb*C *psb*D *psb*E *psb*F *psb*H *psb*I *psb*J *psb*K *psb*L *psb*M *psb*T *psb*Z	15
	Subunits of NADH dehydrogenase	*ndh*A *ndh*B *ndh*C *ndh*D *ndh*E *ndh*F *ndh*G *ndh*H *ndh*I *ndh*J *ndh*K	11
	Subunits of cytochrome b/f complex	*pet*A *pet*B *pet*D *pet*G *pet*L *pet*N	6
	Subunits of ATP synthase	*atp*A *atp*B *atp*E *atp*F *atp*H *atp*I	6
	Large subunit of Rubisco	*rbc*L	1
Self-replication	Large subunits of the ribosome	*rpl14 rpl16 rpl2 rpl20 rpl22 rpl23 rpl32 rpl33 rpl36*	9
	Small subunits of the ribosome	*rps11 rps12 rps14 rps15 rps16 rps18 rps19 rps2 rps3 rps4 rps7 rps8*	12
	DNA-dependent RNA polymerase	*rpo*A *rpo*B *rpo*C1 *rpo*C2	4
	Ribosomal RNAs	*rrn16S rrn23S rrn4.5 rrn5*	4
	Transfer RNAs	*trn*A-UGC *trn*C-GCA *trn*D-GUC *trn*E-UUC *trn*F-GAA *trn*G-GCC *trn*G-UCC *trn*H-GUG *trn*I-GAU *trn*K-UUU *trn*L-CAA *trn*L-UAG *trn*M-CAU *trn*N-GUU *trn*P-UGG *trn*Q-UUG *trn*R-ACG *trn*R-UCU *trn*S-GCU *trn*S-UGA *trn*T-UGU *trn*V-GAC *trn*W-CCA *trn*Y-GUA	24
Other genes	Maturase	*mat*K	1
	Protease	*clp*P1	1
	Envelope membrane protein	*cem*A	1
	C-type cytochrome synthesis gene	*ccs*A	1
	Translation initiation factor	*inf*A	1
Genes of unknown	Proteins of unknown function	*ycf2 ycf3 ycf4*	3

**Figure 3.**
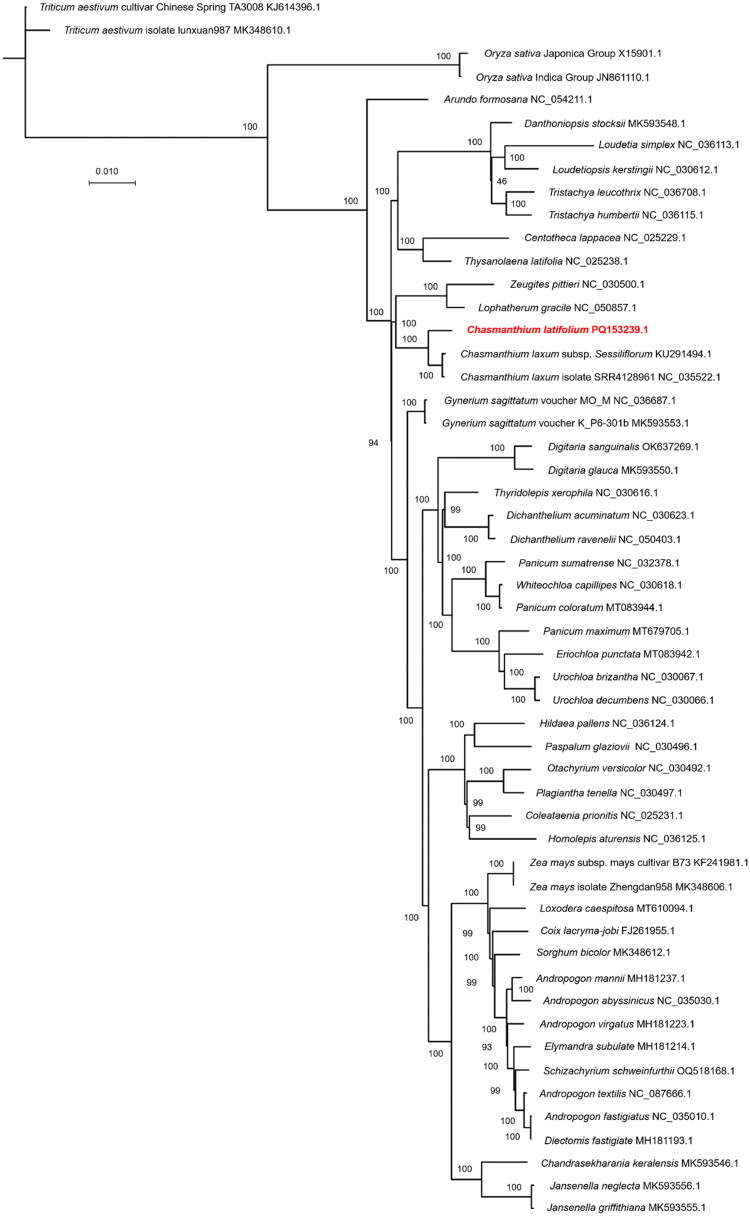
The maximum likelihood (ML) phylogenetic tree was constructed by the complete chloroplast genome of 53 species. *Triticum aestivum* and *Oryza sativa* were used as outgroups. The following sequences were used: *Chasmanthium latifolium* (GenBank:PQ153239.1), *Triticum aestivum* cultivar Chinese Spring TA3008 (GenBank:KJ614396.1; Gornicki et al. 2014), *Triticum aestivum* isolate lunxuan987 (GenBank:MK348610.1; Liu et al. 2019), *Oryza sativa* Japonica Group (GenBank:X15901.1; Morton and Clegg 1993), *Oryza sativa* Indica Group (GenBank:JN861110.1; Zhang et al. 2012), *arundo formosana* (GenBank:NC_054211.1; Feng et al. 2021), *danthoniopsis stocksii* (GenBank:MK593548.1; Bianconi et al. 2020), *loudetia simplex* (GenBank:NC_036113.1; Piot et al. 2018), *loudetiopsis kerstingii* (GenBank:NC_030612.1; Burke et al. 2016), *Tristachya leucothrix* (GenBank:NC_036708.1), *Tristachya humbertii* (GenBank:NC_036115.1; Piot et al.^2018^), *Centotheca lappacea* (GenBank:NC_025229.1; Cotton et al. 2015), *Thysanolaena latifolia* (GenBank:NC_025238.1; Cotton et al. 2015), *Zeugites pittieri* (GenBank:NC_030500.1; Burke et al. 2016), *Lophatherum gracile* (GenBank:NC_050857.1), *Chasmanthium laxum* subsp. *Sessiliflorum* (GenBank:KU291494.1; Burke et al. 2016), *Chasmanthium laxum* isolate SRR4128961 (GenBank:NC_035522.1; Teisher et al. 2017), *Gynerium sagittatum* voucher MO_M (GenBank:NC_036687.1), *Gynerium sagittatum* voucher K_P6-301b (GenBank:MK593553.1), *Digitaria sanguinalis* (GenBank:OK637269.1), *Digitaria glauca* (GenBank:MK593550.1; Bianconi et al.^2020^), *thyridolepis xerophila* (GenBank:NC_030616.1; Burke et al. 2016), *Dichanthelium acuminatum* (GenBank:NC_030623.1; Burke et al. 2016), *Dichanthelium ravenelii* (GenBank:NC_050403.1; Pischl et al. 2020), *Panicum sumatrense* (GenBank:NC_032378.1), *whiteochloa capillipes* (GenBank:NC_030618.1; Burke et al. 2016), *Panicum coloratum* (GenBank:MT083944.1), *Panicum maximum* (GenBank:MT679705.1), *eriochloa punctata* (GenBank:MT083942.1), *Urochloa brizantha* (GenBank:NC_030067.1; Pessoa-Filho et al. 2017), *Urochloa decumbens* (GenBank:NC_030066.1; Pessoa-Filho et al. 2017), *Hildaea pallens* (GenBank:NC_036124.1; Piot et al.^2018^), *Paspalum glaziovii* (GenBank:NC_030496.1; Burke et al. 2016), *Otachyrium versicolor* (GenBank:NC_030492.1; Burke et al. 2016), *Plagiantha tenella* (GenBank:NC_030497.1; Burke et al. 2016), *Coleataenia prionitis* (GenBank:NC_025231.1; Cotton et al. 2015), *Homolepis aturensis* (GenBank:NC_036125.1; Piot et al.^2018^), *Zea mays* subsp. *mays* cultivar B73 (GenBank:KF241981.1; Bosacchi et al. 2015), *Zea mays* isolate Zhengdan958 (MK348606.1), *Loxodera caespitosa* (GenBank:MT610094.1; Welker et al. 2020), *Coix lacryma-jobi* (GenBank:FJ261955.1; Leseberg and Duvall 2009), *Sorghum bicolor* (GenBank:MK348612.1), *Andropogon mannii* (GenBank:MH181237.1; McAllister et al. 2018), *Andropogon abyssinicus* (GenBank:NC_035030.1), *Andropogon virgatus* (GenBank:MH181223.1; McAllister et al. 2018), *Elymandra subulate* (GenBank:MH181214.1; McAllister et al. 2018), *Schizachyrium schweinfurthii* (GenBank:OQ518168.1), *Andropogon textilis* (GenBank:NC_087666.1), *Andropogon fastigiatus* (GenBank:NC_035010.1), *Diectomis fastigiate* (GenBank:MH181193.1; McAllister et al. 2018), *Chandrasekharania keralensis* (GenBank:MK593546.1; Bianconi et al.^2020^), *Jansenella neglecta* (GenBank:MK593556.1; Bianconi et al.^2020^), *Jansenella griffithiana* (GenBank:MK593555.1; Bianconi et al.^2020^). Numbers next to each node indicate bootstrap support values.

The phylogenetic relationships of 53 species were well analyzed in this study using the complete chloroplast genome sequence (Figure 3). *C. latifolium* was most closely related to *C.laxum* (including two plants: *Chasmanthium laxum* subsp. *Sessiliflorum* and *Chasmanthium laxum* isolate SRR4128961), clustered in the same clade. The next most closely related species are *Zeugites pittieri* Hack and *Lophatherum gracile* Brongn, clustered in the same clade.

## Discussion and conclusion

*C. latifolium* is a globally safe species as a popular ornamental plant. In this study, we sequenced and assembled the *C. latifolium* chloroplast genome for the first time, revealing its sequence length of 138,934 bp encoding 105 genes, including the typical quadripartite structure including the SSC and LSC regions separated by two IR regions. The *ycf1* gene has a variety of important functions in the chloroplast genome, which plays a key role in the growth and development of plants, photosynthesis efficiency, and stability of the chloroplast genome. However, previous studies have shown that *ycf1* is not only lacking in grasses and some parasitic plants but also for instance in cranberry (Ericaceae). In this study, the *ycf1* gene was also missing in the chloroplast genome of *C. latifolium*, which further proved that this is a common phenomenon in grasses (de Vries et al. 2015).

Phylogenetic analysis revealed that *C. latifolium*, clustered in a branch with two variants of *C.laxum*(*Chasmanthium laxum* subsp. *Sessiliflorum* and *Chasmanthium laxum* isolate SRR4128961), with the nearest branch of affinity consisting of *Z. pittieri* and *L. gracile*, and the branch consisting of *Centotheca lappacea* (L.) Desv and *Thysanolaena latifolia* (Roxb. ex Hornem.) Honda was next in affinity, which is consistent with the results of a phylogenetic analysis using chloroplast whole genome sequences with one copy of IR removed by Burke et al. 2016. In addition, Sánchez-Ken and Clark conducted a phylogenetic analysis of 56 species in 44 genera using the sequence of *ndh*F, the intron of *rpl16*, and structural data, and the results were also in general agreement with the present study (Sánchez-Ken and Clark 2007). Phylogenetic analyses using the complete chloroplast genome sequence may provide better resolution consistent with morphological descriptions (Lee et al. 2021). The chloroplast genomic data provided in this study may serve as useful information for studying the evolutionary patterns and genetic classification of the genus *Chasmanthium*. The present study searched for phylogeny with 53 plants, including *C. latifolium*, reinforcing previous results in some cases, but also identifying some inconsistencies and the need for more taxonomic sampling of certain genera.

## Supplementary Material

000Supplementary materials.docx

## Data Availability

The genome sequence data supporting this study’s findings are openly available in GenBank of NCBI at (https://www.ncbi.nlm.nih.gov/), reference number PQ153239.1. The associated BioProject, Bio-Sample, and SRA numbers are PRJNA1135130, SAMN42463659, and SRR29813827, respectively.
