# Supplementary material for: Characterization of the complete chloroplast genome of Chasmanthium latifolium (Michx.) H.O.Yates, 1966 (Poaceae)
Source: Mitochondrial DNA B Resour. 2025 Feb 2;10(3):155–61. doi: 10.1080/23802359.2025.2460781 (PMC11792151; doi:10.1080/23802359.2025.2460781)
Supplement: 000Supplementary materials.docx [file TMDN_A_2460781_SM6960.docx]

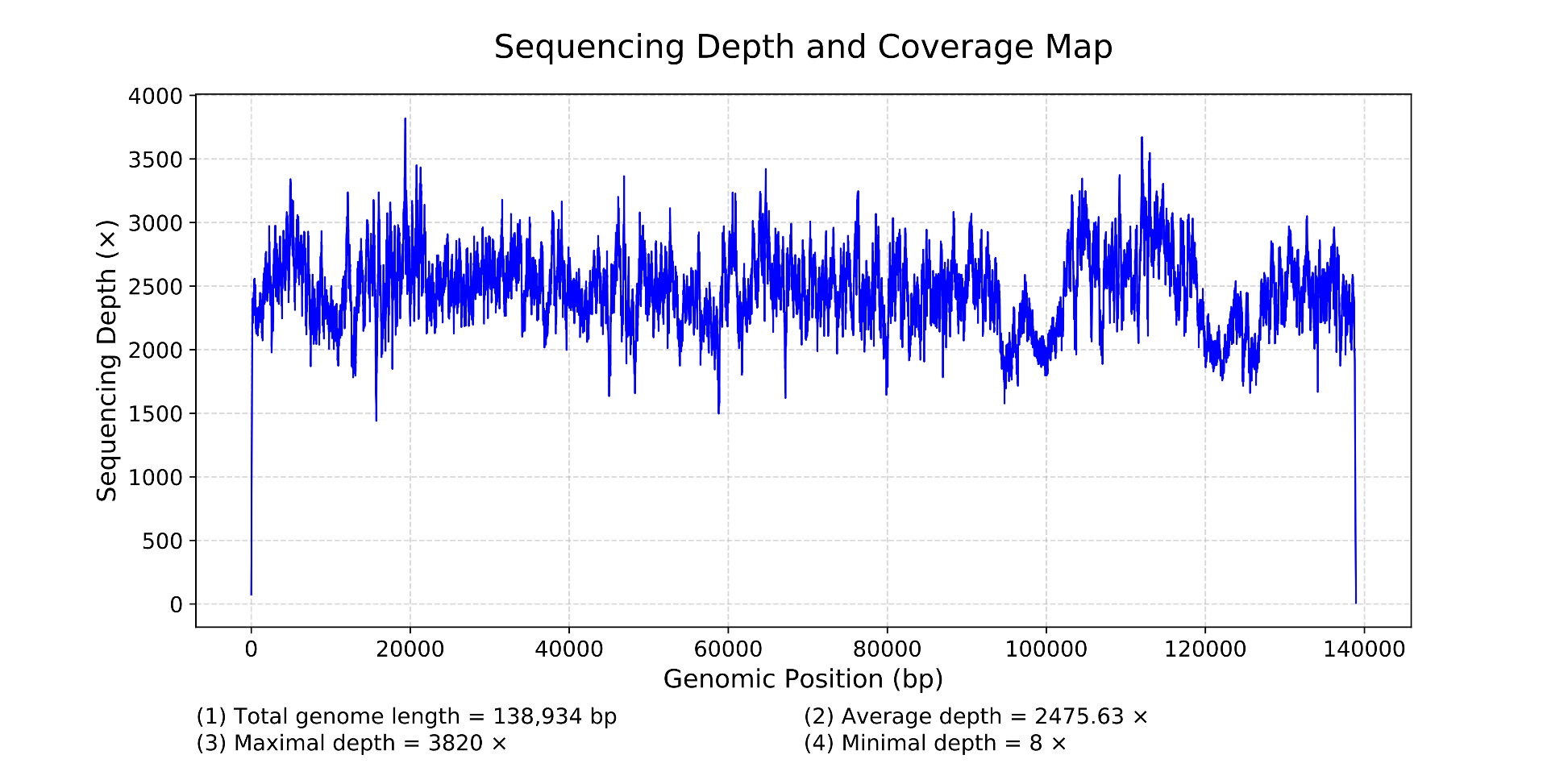
Figure S1. The overall sequencing depth and coverage map


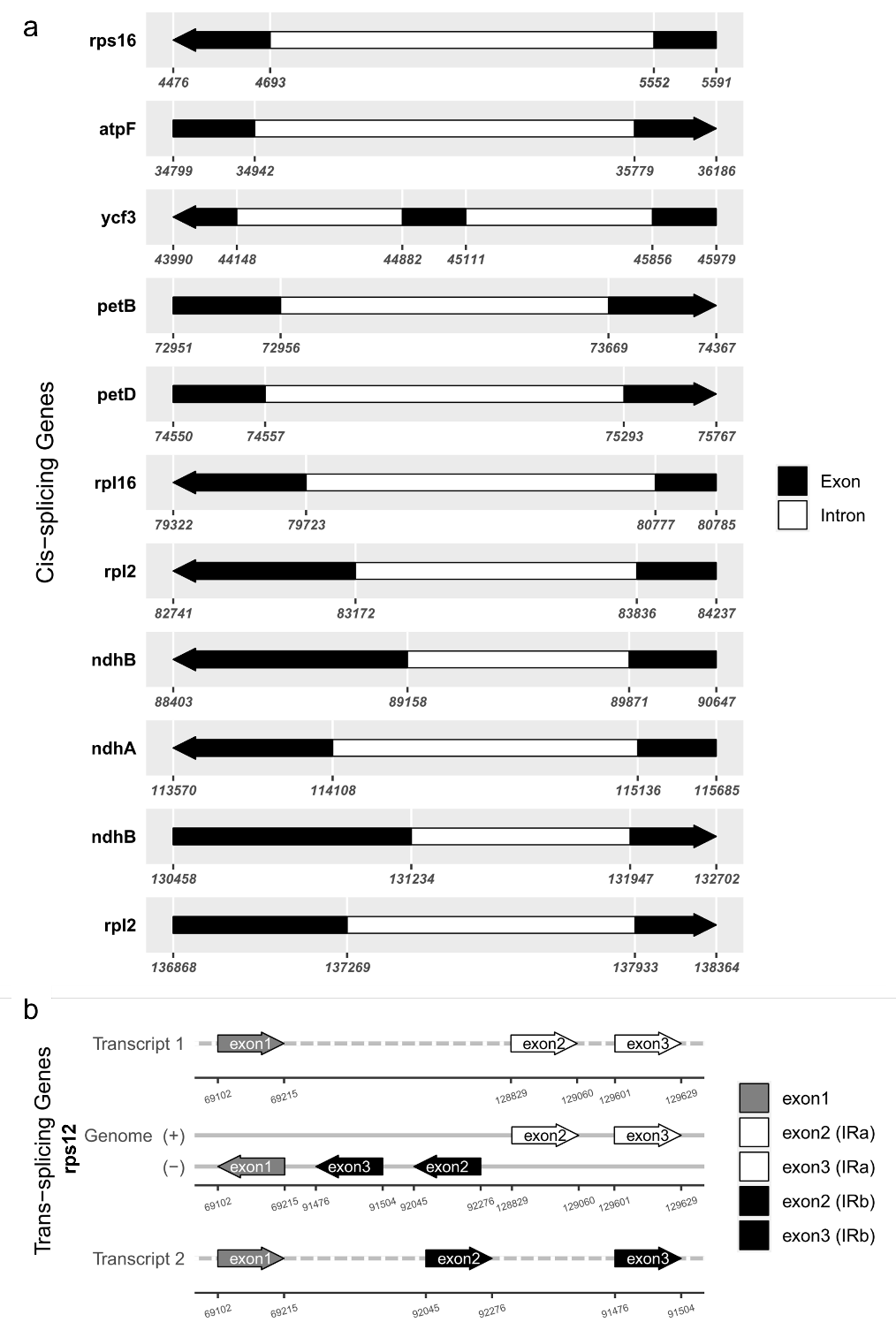


Figure S2. Cis-splicing gene map (a) and trans-splicing gene map (b) visualized in CPGView


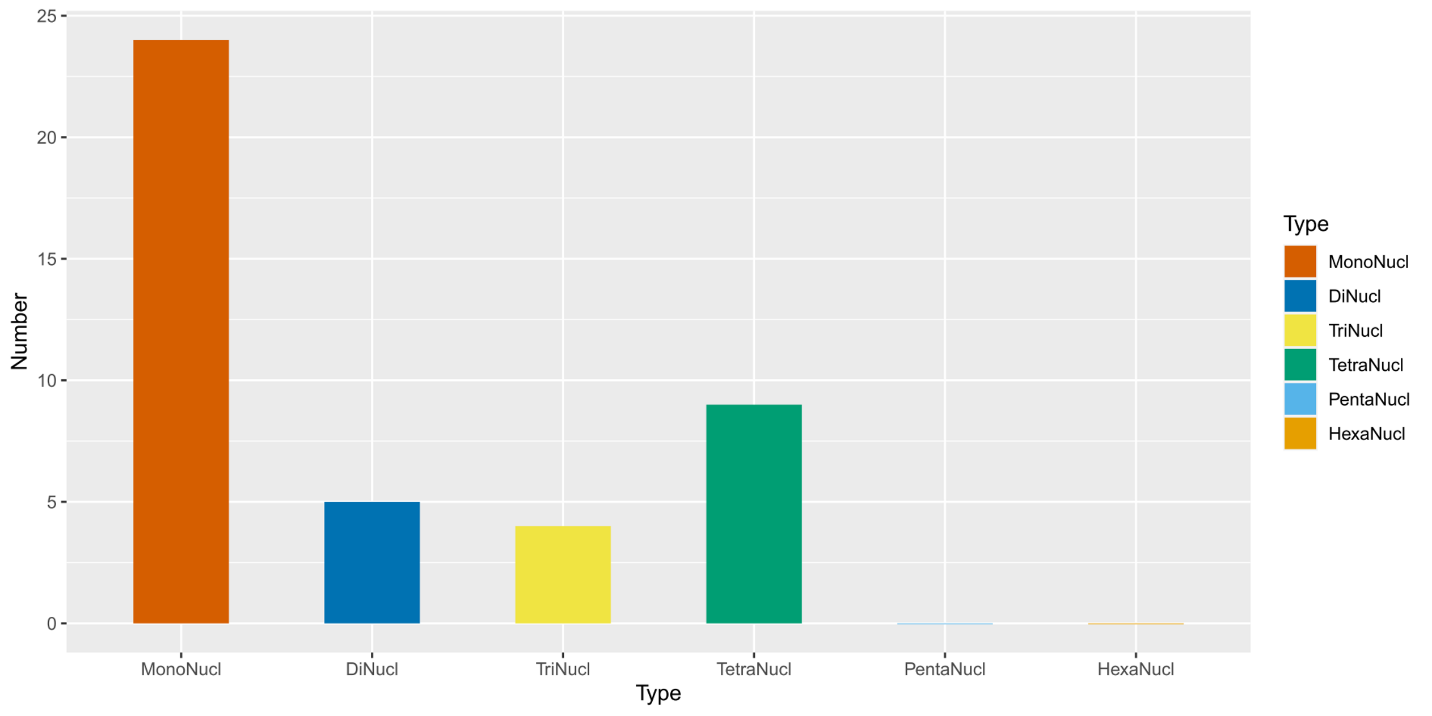
Figure S3. Classification diagram of SSR
